# Appropriateness of Intensive Statin Treatment in People with Type Two Diabetes and Mild Hypercholesterolemia: A Randomized Clinical Trial

**DOI:** 10.34172/aim.2023.45

**Published:** 2023-06-01

**Authors:** Mohammad Taghi Gorji, Fariba Alaei-Shahmiri, Gisoo Darban Hosseini Amirkhiz, Seyed Hashem Sezavar, Mojtaba Malek, Mohammad E Khamseh

**Affiliations:** ^1^Endocrine Research Center, Institute of Endocrinology and Metabolism, Iran University of Medical Sciences (IUMS), Tehran, Iran; ^2^Research Center for Prevention of Cardiovascular Disease, Institute of Endocrinology and Metabolism, Iran University of Medical Sciences (IUMS), Tehran, Iran

**Keywords:** High-intensity statin, Hyperlipidemia, LDL, Moderate-intensity statin, Type 2 diabetes

## Abstract

**Background:**

The aim of this study was to compare moderate- versus high-intensity statin therapy in patients with type 2 diabetes and low-density lipoprotein (LDL) cholesterol less than 130 mg/dL.

**Methods:**

This was a randomized, open-label, parallel design trial comprised of 79 patients randomly allocated into two groups receiving high-intensity [atorvastatin 40 mg (A40) or rosuvastatin 20 mg (R20) daily] or moderate-intensity [atorvastatin 20 mg (A20) or rosuvastatin 10 (R10) mg daily] statins for eight weeks. The variables investigated were lipid profile, high sensitivity C-reactive protein (hs-CRP), and interleukin-6 (IL-6).

**Results:**

The percentage of decrease in LDL levels (±SD) for the high-intensity group (-35.5±25.5) was significantly greater than the moderate-intensity group (-24.6±23.5) (*P*=0.04). While 38.1% (n:8) of patients receiving A20 and 55% (n:11) of those being on R10 achieved the targets of≥30% reduction in the LDL level, these figures were 63.2% (n=12) and 73.8% (n=14) for A40 and R20 subgroups, respectively. Subsequently, the likelihood of achieving LDL reduction≥30%, was significantly greater with high-intensity statin therapy (OR: 3.1, 95% CI: 1.09, 8.90, *P*=0.03). Logistic regression analysis also showed that for every 1 mg/dL increase in the baseline LDL level, the odds of achieving the LDL reduction≥30% increased by 1.04 times [95% CI: (1.01, 1.07), *P*=0.003].

**Conclusion:**

Despite the general conception, moderate-intensity statins are not adequate for the majority of patients with T2DM and mild hyperlipidemia and greater numbers of patients could reach the LDL cholesterol target with high-intensity statin therapy.

## Introduction

 Atherosclerosis remains the leading cause of mortality in human beings.^1^ Dyslipidemia has an important role in the development of the atherosclerotic disease.^2^ The prevalence of dyslipidemia is greater among patients with type 2 diabetes mellitus (T2DM).^3^ A high level of low-density lipoprotein (LDL) cholesterol is one of the most important risk factors for cardiovascular problems.^4^ Statins are considered as the first-line medical treatment for prevention of atherosclerotic cardiovascular disease (ASCVD). The intensity of statin therapy depends on age, duration of DM, LDL level, clinical presentations, and ASCVD risk. The aim of moderate-intensity statin therapy is 30%-50% and high-intensity 50% or more reduction in LDL level. Although both treatments can decrease the risk of ASCVD, the greater the LDL reduction, the lower the risk.^5^

 According to the baseline features, different intensity of statin treatment is necessary to treat LDL cholesterol to the target.^6^ The strategy used to prevent over- or under- treatment is titration,^7^ which, although an effective and accurate method, is costly and time-consuming. It has been reported that a large number of patients do not achieve the therapeutic goals.^8,9^ Under-treatment may not reduce the ASCVD risk to the optimum. On the other hand, over-treatment raises the costs and increases the risk of side effects such as myopathy, liver dysfunction, and elevated risk of diabetes, which are dose-dependent.^10^

 In this study, we aimed to survey the effects of different intensity statin therapies on LDL level in patients with T2DM and mild hyperlipidemia.

## Materials and Methods

###  Subjects

 The study consisted of patients aged 40 to 75 years with T2DM and mild hyperlipidemia who did not have ASCVD and were recommended to take moderate-intensity statin therapy for primary prevention according to the ADA 2018 guideline^7^ and had medical record at the Institute of Endocrinology and Metabolism, Iran University of Medical Sciences (IUMS). Pregnant or lactating women, patients already on lipid-lowering agents (statins, bile acid binding resins, cholesterol absorption inhibitor, fibrates, niacin, omega-3 fatty acids), those with genetic disorders, renal failure, rheumatic diseases, untreated thyroid disorders, biliary or liver diseases, as well as individuals with elevated levels of serum alanine aminotransferase (ALT > 3 ULN) or creatine phosphokinase (CPK > 10 ULN), patients on corticosteroids, cyclosporins or hormone replacement therapy, history of alcohol use, acute and chronic infectious or inflammatory disease were excluded from the study.

###  Study Design and Procedure

 This study was a randomized, open-label, parallel design trial carried out between November 2019 and July 2020. Eligible patients were randomly assigned to the study groups receiving high-intensity (atorvastatin 40 mg or rosuvastatin 20 mg daily) or moderate-intensity (atorvastatin 20 mg or rosuvastatin 10 mg daily) statins for eight weeks by performing block randomization with a block size of 4.

 The primary objective was to compare the effects of moderate- and high-intensity statin therapies on LDL cholesterol. The secondary objective included the effects of intensity of treatment on high-sensitivity C-reactive protein (hs-CRP) and interleukin-6 (IL-6).

 This study was reviewed and approved in two subsets by Iran University of Medical Science’s Institutional Review Board and was registered in the clinical trials database. The research protocol is available online (https://www.irct.ir; identifier: IRCT20180929041169N1; date: 07/01/2019 and identifier: IRCT20180929041169N2; date: 10/01/2019). The study was conducted according to the Declaration of Helsinki and was approved by the ethics committee of Iran University of Medical Sciences (Approval number IR.IUMS.FMD.REC.1398.375). Informed consent was obtained from all participants prior to enrollment. The Abidi Pharmaceuticals had supplied the medications (atorvastatin and rosuvastatin). This study follows the recommendations proposed by the CONSORT Statement.

###  Clinical Measurements

 Demographic, social and medical history of participants, including history of smoking, hypertension, other diseases and drug consumption were obtained. The patients’ weight and height were measured and body mass index (BMI) was calculated as follows: BMI = weight (kg)/[height(m)]^2^. The systolic blood pressure (SBP) and diastolic blood pressure (DBP) were measured by an experienced nurse using a manual brachial sphygmomanometer with patients in a sitting position, after five minutes of rest while their arm was positioned at their heart level. The average of three measurements was reported. Patients on antihypertensive treatment and those with SBP ≥ 140 mm Hg or DBP ≥ 90 mm Hg were considered as having hypertension (HTN). Diabetes was diagnosed based on the American Diabetes Association’s guidelines or previous history of diabetes. Individuals with a history of at least 100 cigarettes in their lifetime and currently smoking were considered as current smokers.

###  Laboratory Examination

 Blood samples were collected after an overnight fasting of at least 8 hours. Fasting blood glucose (FBS), triglyceride (TG), total cholesterol, high-density lipoprotein (HDL), LDL, aspartate transaminase (AST), ALT, and CPK were measured by standard enzymatic method with Pars Azmun diagnostic kits (Pars Azmun Co., Tehran, Iran). The intra- and inter-assay coefficients of variation were respectively 1.5 and 0.8 for FBS, 1.5 and 1.1 for TG, 0.7 and 1.3 for HDL, 0.6 and 1.3 for LDL, 3.1 and 1.4 for AST, 2.7 and 1.6 for ALT, and 1.5 and 1.1 for CPK. IL-6 and hs-CRPand were measured with the chemiluminescent immunometric method using an IMMULITE 2000 immunometric assay system (Siemens Healthcare GmbH, Erlangen, Germany).

###  Statistical Methods

 The data were analyzed using IBM SPSS Statistics for Windows (Version 22.0 IBM Corp. Released 2013. Armonk, NY). Continuous variables are expressed as mean ± SD, or as median (IQR) for skewed data. Categorical variables are presented as n (% within group). Within-group comparisons were performed using a paired-samples *t* test or a Wilcoxon test for normally distributed and non-normal data, respectively. Variables of interest were compared between the treatment groups using χ^2^ test, analysis of variance (ANOVA), analysis of covariance (ANCOVA) or a non-parametric test, as appropriate. Moreover, the logistic regression models were fitted to evaluate the effects of treatments and other covariates on the dichotomous responder outcomes, including patients achieving the treatment goals of: ≥ 30% and ≥ 50% reduction in cholesterol, LDL-C and TG levels; ≥ 30% and ≥ 50% increase in HDL; and ≥ 25% reduction in inflammatory markers of hs-CRP and IL-6. We explored the impact of statin therapies on serum cholesterol and LDL based on the intensity of the treatments. In these analyses, participants were categorized into two groups: 1) those treated with the moderate-intensity atorvastatin (20 mg/d) or rosuvastatin (10 mg/d), and 2) participants who received the high-intensity statin treatments (40 mg/d atorvastatin or 20 mg/d rosuvastatin). All tests were 2-tailed, and *P* ≤ 0.05 was considered statistically significant.

 Sample size was calculated based on a predicted 20% (30 mg/dL) reduction in serum LDL level after statin therapy, assuming a standard deviation of 30 mg/dL.^11^ Using a clinical trial formula,^12^ a sample of 72 participants (18/subgroup) could provide sufficient power (85%) to detect the expected changes at the 5% significance level. Ninety-nine patients were recruited to allow for drop out/non-compliance.

## Results

**Figure 1:**
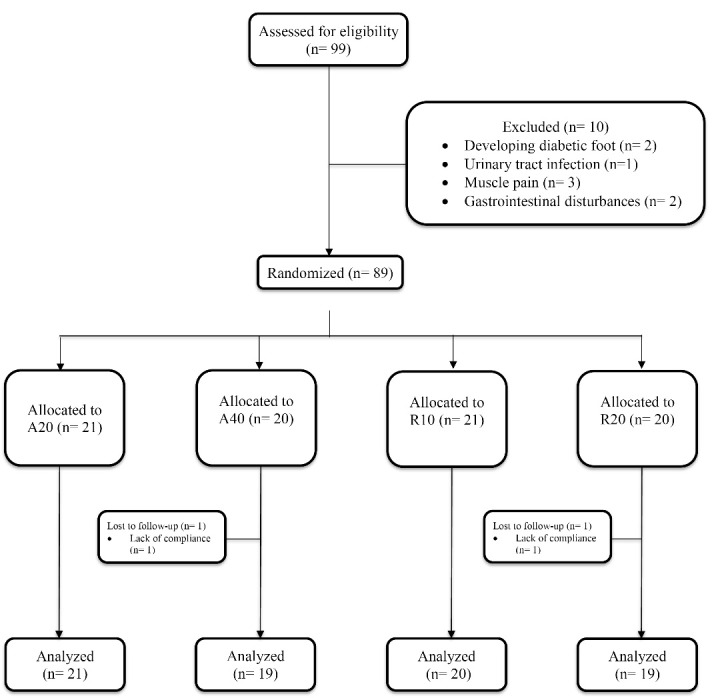
Consolidated Standards of Reporting Trials (CONSORT) Diagram for Patient Enrolment, Follow-up, and Analysis of the Treatment Outcome. A20, atorvastatin 20 mg/d; A40, atorvastatin 40 mg/d; R10, rosuvastatin 10 mg/d; R20, rosuvastatin 20 mg/d

 Of the 99 patients starting the study, data of 79 participants (38 men and 41 women) with a mean ( ± SD) age of 55.7 ± 9.1 years who completed the study were used for final analysis (Figure 1). As presented in Table 1, the four treatment groups were comparable in terms of age, gender and clinical characteristics at baseline (*P* values > 0.05).

**Table 1:** Baseline Characteristics of the Participants.

**Variables**	**Atorvastatin 20 mg (n=21)**	**Atorvastatin 40 mg (n=19)**	**Rosuvastatin 10 mg (n=20)**	**Rosuvastatin 20 mg (n=19)**	***P *****Value**
Age (y)	56.0 ± 9.5	56.6 ± 8.7	55.2 ± 10.1	53.6 ± 7.2	0.73
Female (%)	11 (52.4%)	10 (52.6%)	11 (55.0%)	9 (47.4%)	0.971
BMI (kg/m^2^)	26 (24, 28)	28 (25, 30)	26 (24, 30)	26 (24, 28)	0.229
HTN	7 (33.3%)	5 (26.3%)	6 (30.0%)	6 (31.6%)	0.969
SBP > 140 mm Hg	3 (14.3%)	1 (5.3%)	1 (5%)	2 (10.5%)	0.681
DBP > 90 mm Hg	0 (0%)	0 (0%)	0 (0%)	0 (0%)	1.000
Current Smokers (%)	7 (33.3%)	4 (21.1%)	4 (20.0%)	6 (31.6%)	0.688
Duration of DM (y)	10 (6, 15)	7 (3, 8)	6.5 (4, 10.5)	3 (3, 8)	0.160
Insulin Therapy (%)	11 (52.4%)	12 (63.2%)	10 (50.0%)	6 (31.6%)	0.269
FBS (mg/dL)	124 (112, 145)	152 (120, 185)	132 (97, 187)	130 (99, 187)	0.309
TG (mg/dL)	153 (102, 176)	135 (111, 172)	120 (65, 165)	102 (84, 155)	0.592
Cholesterol (mg/dL)	160 (132, 198)	164 (140, 200)	169 (149, 178)	163 (154, 196)	0.650
LDL (mg/dL)	93.86 ± 28.65	93.21 ± 26.78	92.75 ± 25.87	94.06 ± 20.76	0.999
HDL (mg/dL)	43.1 ± 10.0	42.6 ± 10.6	48.5 ± 12.2	45.7 ± 8.5	0.234
Cr (mg/dL)	1 (0.85, 1.1)	0.9 (0.8, 1.1)	1 (0.9, 1.2)	1 (0.9, 1)	0.200
hs-CRP (mg/dL)	3.2 (1.7, 5.6)	4.0 (2.8, 4.9)	2.3 (1.3, 5.4)	2.1 (1.4, 4.6)	0.361
IL-6 (pg/mL)	3.3 (2.1, 4.3)	3.2 (2.3, 4.3)	3.2 (2.3, 4.4)	3.4 (2.4, 4.1)	0.800

Continuous variables are expressed as mean ± SD or as median (IQR) for skewed data. Categorical variables are presented as n (% within group). Between-group comparisons were performed using χ^2^ test, ANOVA or a nonparametric test (Median test), as appropriate.

###  Effect of Statin Therapies on Serum lipids & Inflammatory Markers 

**Figure 2:**
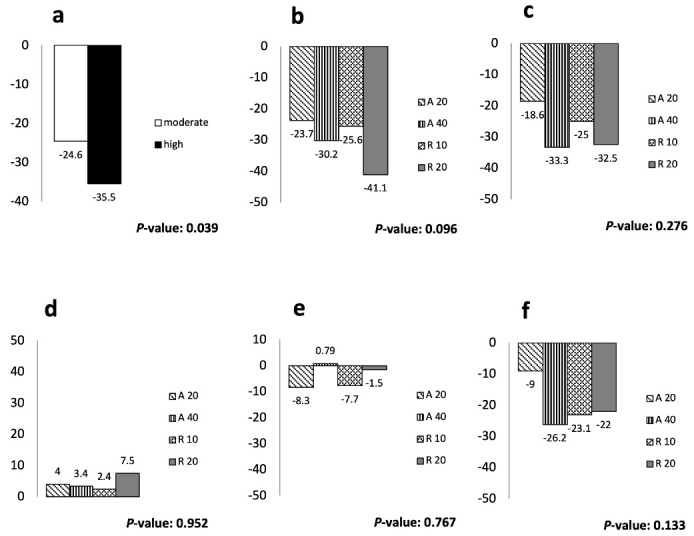
Percentage of Change from the Baseline Value in Serum Lipids by the Treatment Group. (**a)** Mean change in LDL levels (%); (**b)** mean change in LDL levels (%); (**c)** mean change in non-HDL levels (%); (**d)** Mean changes in HDL levels (%); **(e)** median changes in TG levels (%); (**f)** median change in cholesterol levels (%). Between group comparisons were performed using ANOVA or non-parametric tests (median test), as appropriate. LDL, low-density lipoprotein cholesterol; HDL, high-density lipoprotein cholesterol; TG, triglyceride; A20, atorvastatin 20 mg/d; A40, atorvastatin 40 mg/d; R10, rosuvastatin 10 mg/d; R20, rosuvastatin 20 mg/d; low-dose, atorvastatin 20 mg and rosuvastatin 10 mg; high-dose, atorvastatin 40 mg and rosuvastatin 20 mg

 Statin therapy for eight weeks decreased the mean ( ± SD) LDL levels of participants receiving moderate-intensity statins (from 93.32 ± 26.99 to 67.59 ± 24.09 mg/dL, *P* < 0.001) as well as those on high-intensity statins (from 93.62 ± 23.73 to 58.35 ± 22.50 mg/dL, *P* < 0.001). Although the decline in LDL levels was greater for the high-intensity group, the between-group difference was statistically borderline (*P* = 0.06) (Table 2). Also, the percentage of decrease in LDL levels ( ± SD) for the high-intensity group (-35.5 ± 25.5) was significantly greater than the moderate intensity statins (-24.6 ± 23.5) (*P* = 0.04; Figure 2). There were no significant differences in between-subgroup comparisons in terms of the LDL lowering effect (*P* = 0.17) (Table 3). Similarly, total cholesterol and non-HDL cholesterol levels measured after eight weeks were significantly lower than baseline in both groups and all four subgroups; however, no meaningful change was detected in HDL or TG levels within- or between- groups or subgroups (Tables 2 and 3).

**Table 2:** Effects of Statin Therapy on Serum Lipids & Inflammatory Markers Stratified by Treatment Intensity

**Markers**	**Moderate intensity (n=41)**				**High intensity (n=38)**				**Between- group comparison**	
	**Baseline**	**Week 8**	**Changes**	***P***	**Baseline**	**Week 8**	**Changes**	***P***	Difference of the changes*	***P***
LDL (mg/dL)	93.32 ± 26.99	67.59 ± 24.09	-25.73 ± 25.29	< 0.001	93.62 ± 23.73	58.35 ± 22.50	-35.27 ± 27.06	< 0.001	9.35 (-0.23, 18.94)	0.056
Chol (mg/dL)	166 (143.5,183.5)	131 (108,160)	-17 (-53, -1)	< 0.001	163.5 (143.5,198.5)	117.5 (102.25,157)	-34.5 (-65, -17)	< 0.001	8.42 (-5.92, 22.77)	0.246
TG (mg/dL)	146 (91.5,172)	119 (84.5,158.5)	-6 (-51, 14)	0.07	131 (89.75,167.5)	118 (96,149.5)	0 (-39, 19)	0.35	-7 (-29, 15)	0.444
HDL (mg/dL)	45.93 ± 11.23	47.20 ± 10.75	1.27 ± 8.68	0.355	44.24 ± 9.67	46.61 ± 9.51	2.37 ± 9.42	0.130	0.42 (-3.20, 4.04)	0.818
Non-HDL (mg/dL)	119.34 ± 38.12	89.56 ± 34.77	-29.78 ± 35.94	< 0.001	126.82 ± 29.25	84.66 ± 39.06	-42.16 ± 36.23	< 0.001	8.83 (-5.81, 23.47)	0.233
IL-6 (pg/mL)	3.30 (2.25,4.35)	2.30 (2.05,2.85)	-0.6 (-1.5, 0)	< 0.001	3.25 (2.38,4.15)	2.30 (2.08,2.60)	-0.7 (-1.6, -0.1)	< 0.001	0.1 (-0.3, 0.7)	0.495
hs-CRP (mg/L)	2.60 (1.45,5.35)	2.10 (1.3,3.45)	-0.7 (-2, 0.5)	0.024	3.65 (1.58,4.82)	1.85 (0.98,4.38)	-1 (-3.2, 0.7)	0.021	0.2 (-0.9, 1.4)	0.662

Continuous variables are expressed as mean ± SD or as median (IQR) for skewed data unless otherwise stated. Within-group comparisons were performed using a paired sample t-test or a Wilcoxon test for normally distributed and non-normal data, respectively. Between-group comparisons were performed using ANCOVA (adjusted for baseline values) or a nonparametric test (Mann-Whitney test), as appropriate. *Data are presented as mean or median (95% CI).

**Table 3:** Effects of Statin Therapy on Serum Lipids & Inflammatory Markers Stratified by Statin Types and Dosages.

**Markers**	**Moderate-intensity**				**High-intensity**				***P***
	**A20 Group (n=21)**		**R10 Group (n=20)**		**A40 Group (n=19)**		**R20 Group (n=19)**		
	**Baseline**	**Week 8**	**Baseline**	**Week 8**	**Baseline**	**Week 8**	**Baseline**	**Week 8**	
LDL (mg/dL)	93.9 ± 28.7	69.4 ± 29.0^c^	92.8 ± 25.9	65.7 ± 18.2^c^	93.2 ± 26.8	61.8 ± 23.2^c^	94.1 ± 20.8	54.6 ± 21.7^c^	0.17
Chol. (mg/dL)	160 (132,198)	132 (113, 172)^b^	169(149,178)	129 (107, 149)^b^	164(140,200)	118 (103, 143)^b^	163 (154,196)	117 (100, 161)^b^	0.30
TG (mg/dL)	153 (102,176)	137 (98,160)	118 (66,164)	94 (77,130)	135(111,172)	127 (97,176)	102 (84,155)	118 (76,140)	0.77
HDL (mg/dL)	43.2 ± 10.0	44.20 ± 9.3	48.8 ± 11.9	50.4 ± 11.5	42.7 ± 10.7	45.2 ± 10.0	45.8 ± 8.6	48.0 ± 9.1	0.69
Non-HDL (mg/dL)	123.1 ± 41.6	96.3 ± 36.3^b^	115.5 ± 34.7	82.5 ± 32.5^c^	127.4 ± 30.5	83.9 ± 37.7^c^	126.2 ± 28.8	85.4 ± 41.4^c^	0.50
IL-6 (pg/mL)	3.3(2.1,4.3)	2.3(2.0,3.4)	3.2(2.3,4.4)	2.3(2.1,2.6)^b^	3.2(2.3,4.3)	2.3(2.1,2.6)^b^	3.4(2.4,4.1)	2.3(2.0,2.7)^b^	0.65
hs-CRP (mg/L)	3.2 (1.7,5.6)	2.1 (1.3,3.4)	2.3(1.3,5.4)	2.0(0.87,3.4)	4 (2.8,4.9)	1.8 (1,4.6)^a^	2.1(1.4,4.6)	1.9 (0.9,4.2)	0.47

Continuous variables are expressed as mean ± SD or as median (IQR) for skewed data. Within-group comparisons were performed using a paired-samples t-test or a Wilcoxon test for normally distributed and non-normal data, respectively. Between-group comparisons were performed using ANCOVA (adjusted for baseline values) or a nonparametric test (Median test), as appropriate. ^a^*P* < 0.05 compared to baseline; ^b^*P* < 0.01 compared to baseline; ^c^*P* < 0.00.

 Eight-week statin therapy also resulted in significantly reduced level of hs-CRP in both moderate- and high-intensity groups (*P* = 0.024 and *P*= 0.021,respectively) without a significant difference between the groups. A similar effect was observed for IL-6 levels. The statin-induced changes in these inflammatory markers did not differ significantly across the four treatment subgroups (Table 2).

###  Effects of Statin Therapy on the Responder Outcomes 

 Responder outcomes for lipid profile were evaluated as the percentage of patients reaching the targets of ≥ 30% and ≥ 50% change in LDL, total cholesterol, TG, HDL, and non-HDL for each group. The proportion of patients achieving the targets of ≥ 30% decrease in the LDL level was 38.1% (8 patients) for the A20 subgroup, 55% (11 patients) for the R10, 63.2% (12 patients) for the A40, and 73.8% (14 patients) for the R20. These figures for the targets of ≥ 50% decline in LDL levels ranged between 19% and 42.1% for the A20 and R20 subgroups, respectively. Nineteen percent of patients in the A20 subgroup and 30% of the R10 subgroup as well as 42.1% of the A40 and R20 subgroups reached the target of ≥ 30% decrease in total cholesterol (Table 4). There was a significant difference between the proportions of participants in the high-intensity group who achieved the LDL goal of ≥ 30% and those in the moderate-intensity group (70.3% vs. 46.3%, *P* = 0.03), but the difference was non-significant for ≥ 50% LDL reduction (37.8% vs. 19.5%, *P* = 0.07). Moreover, a numerically greater proportion of patients treated with high-intensity statin achieved the specified cholesterol target of ≥ 30% reduction compared to those on moderate-intensity statin therapy (42.1% vs. 24.4%, *P* = 0.09).

**Table 4:** Proportion of Patients Achieving the Lipids and Inflammatory Markers Targets Stratified by Treatment Intensity

**Responder Outcomes**	**Moderate-intensity**		**High-intensity**	
	**A20 (n=21)**	**R10 (n=20)**	**A40 (n=19)**	**R20 (n=19) **
Patients achieving LDL targets, n (%)				
≥ 30% decrease	8 (38.1%)	11 (55.0%)	12 (63.2%)	14 (73.8%)
≥ 50% decrease	4 (19.0%)	4 (20.0%)	6 (31.6%)	8 (42.1%)
Patients achieving chol targets, n (%)				
≥ 30% decrease	4 (19.0%)	6 (30.0%)	8 (42.1%)	8 (42.1%)
≥ 50% decrease	2 (9.5%)	0 (0.0%)	0 (0.0%)	0 (0.0%)
Patients achieving TG targets, n (%)				
≥ 30% decrease	5 (23.8%)	5 (25.0%)	4 (21.1 %)	5 (26.3 %)
≥ 50% decrease	1 (4.8 %)	4 (20.0 %)	2 (10.5 %)	1 (5.3 %)
Patients achieving HDL targets, n (%)				
≥ 30% decrease	3 (14.3 %)	2 (10.0 %)	3 (15.8 %)	2 (10.5 %)
≥ 50% decrease	0 (0%)	0 (0%)	1 (5.3 %)	0 (0%)
Patients achieving non-HDL targets, n (%)				
≥ 30% decrease	6 (28.6 %)	12 (60.0 %)	12 (63.2 %)	11 (57.9 %)
≥ 50% decrease	3 (14.3 %)	3 (15.0 %)	5 (26.3 %)	7 (36.8 %)
Patients achieving hs-CRP reduction of ≥ 25%, n (%)	9 (42.9 %)	10 (50.0 %)	10 (52.6 %)	10 (52.6 %)
Patients achieving IL-6 reduction of ≥ 25%, n (%)	8 (38.1 %)	9 (45.0 %)	9 (47.4 %)	9 (47.4 %)

 Responder outcomes for inflammatory markers were evaluated as percentage of patients reaching the target of ≥ 25% decrease in hs-CRP and IL-6 for each subgroup. The highest success rate pertained to the A40 and R20 subgroups with 52.6% of patients reaching the target for hs-CRP. The lowest success rate pertained to the A20 subgroup in which 38.1% of patients reached the target for IL-6 (Table 4).

 Subsequent logistic regression analysis showed the administrated statin therapy as a significant determinant for achieving the LDL target of ≥ 30% reduction, with more than twice greater odds among patients treated with high-intensity statins compared to those on moderate-intensity [OR: 3.11, 95% CI: (1.08, 8.89), *P* = 0.034]. Additionally, we found that the odds of achieving the LDL target of ≥ 30% reduction decreased with increasing BMI and each 1 kg/m^2^ increase in BMI led to a 15% decline in the chance of attaining the target [OR: 0.85, 95% CI: (0.73, 0.99), *P* = 0.038]. Multivariate analyses also highlighted a direct association between the baseline LDL and achieving LDL goal of ≥ 30% decrease. Each 1 mg/dL increase in baseline LDL level increased the chance of achieving the target by 4% [OR: 1.04, 95% CI: (1.01, 1.07), *P* = 0.003] (Table 5).

**Table 5:** Logistic Regression Analyses Comparing the Main Predictors of Achieving LDL Target ≥ 30% Decrease

	**Parameters**	**B**	**SE**	***P***** Value**	**OR**	**95% CI for OR**	
						**Lower**	**Upper**
Model 1	Treatment groups						
	Moderate-Intensity (Ref)	—					
	High-intensity	1.135	0.536	**0.034**	3.11	1.088	8.898
	BMI (kg/m^2^)	-0.164	0.079	**0.038**	0.849	0.727	0.991
	Baseline LDL (mg/dL)	0.038	0.013	**0.003**	1.039	1.013	1.066
	Age (year)	-0.061	0.033	0.068	0.941	0.882	1.005
Model 2	Treatment groups						
	A 20 (Ref)	—					
	A 40	1.465	0.771	0.057	4.330	0.956	19.616
	R 10	0.978	0.734	0.182	2.660	0.631	11.205
	R 20	1.905	0.813	**0.019**	6.717	1.366	33.035
	BMI (kg/m^2^)	-0.165	0.081	**0.041**	0.848	0.724	0.993
	Baseline LDL (mg/dL)	0.041	0.014	**0.003**	1.041	1.014	1.070
	Age (y)	-0.063	0.034	0.069	0.939	0.878	1.005

Dependent variable: achievement the target of ≥ 30% reduction in LDL levels; Model 1: multivariate regression model with the study groups categorized based on the treatment intensity; Moderate-intensity group includes those receiving A20 or R10; High-intensity group includes those receiving A40 or R20; Model 2: multivariate logistic regression model with the study groups categorized based on different statin types and dosages; A20, atorvastatin 20 mg; A40, atorvastatin 40 mg; R10, rosuvastatin 10 mg; R20, rosuvastatin 20 mg; BMI, body mass index; LDL, low-density lipoprotein cholesterol. *P* values that are less than 0.05 are in bold.

## Discussion

 We found that high-intensity statin therapy increased the chance of achieving the LDL target in people with type 2 diabetes and mild hyperlipidemia. Also, both moderate- and high-intensity statin therapies could significantly reduce the hs-CRP and IL-6 levels in this population.

 Isolation of substances from fungi with the ability to impair the activity of HMG-CoA reductase, which is an essential enzyme for cholesterol production, led to the emergence of the most important cholesterol lowering drugs.^13^ Inhibition of the mentioned enzyme reduces the hepatic cholesterol accumulation and finally ends up in up-regulation of LDL receptors in the liver. This is the mechanism by which statins reduce the LDL level.^14^ Each 1 mmol/L (38.5 mg/dL) reduction in LDL level after the first year of statin consumption results in about a 25% decrease in major cardiovascular events.^15^ Established vital benefits, relatively low cost, and minimum adverse effects made them the first-line medical treatment for primary and secondary prevention of ASCVD.^16^

 Moderate- and high-intensity statin therapies are expected to decrease the LDL level by ≥ 30% and ≥ 50%, respectively. However, even after administration of the highest doses of statins, there are a number of patients who still cannot meet the goal.^17^ A meta-analysis compared the variability of responses to statin use in a large number of patients. In that study, the percentage of patients who failed to reach the LDL target was less than our results in all four treatment subgroups. Namely, 61.9% of patients in our study versus 12.8% in the study by Karlson et al failed to reach ≥ 30% reduction in LDL after daily 20 mg atorvastatin use.^17^ Only 29% of patients were diabetic in that study and the mean baseline LDL level was significantly higher than our population, which could be the reasons for the observed differences.

 All four drugs were successful in lowering LDL; however, the effect of rosuvastatin is more powerful in comparison to other statins.^18^ This finding is consistent for patients with diabetes.^19^ According to a meta-analysis of 75 randomized control trials, prescribing a daily dose of 20 mg atorvastatin or 10 mg rosuvastatin can decrease LDL levels by more than 40%.^20^ Although our findings emphasize the higher potency for rosuvastatin, the overall drug potency for statins was smaller than previous studies and this observation could be due to the relatively lower baseline LDL level in our study population.^21,22^ Besides, in a population consisting of patients with T2DM, the increased production of very low density lipoprotein (VLDL) in the liver resulting from elevated insulin resistance can interfere with the lipid-lowering ability of the statins.^23,24^

 There are also genetic variations in apolipoprotein E locus which lead to different responses to statins in terms of their LDL lowering effect.^25,26^ Furthermore, drug pharmacokinetics are not the same in different populations^27^ and it is shown that higher doses of statins are required for Westerners to reach the same percentage of LDL decline compared to East Asian people.^28^ Thus, the observed lower efficacy for statins in our study could also be the consequence of the distinct genotype of the study population.

 We found that BMI is a confounder for LDL target achievement in people with T2DM and mild hyperlipidemia. The chance of achieving the LDL goal decreased with increasing BMI. It is well-known that obesity alters drug pharmacokinetics and lipid metabolism.^29^ Dyslipidemia is more prevalent among obese individuals.^30^ A cross-sectional study investigating the impact of obesity and DM on the LDL therapeutic goal attainment observed that obesity and DM independently predicted failure to reach the LDL goal. According to the receiver operating characteristic (ROC) analysis, individuals with BMI ≥ 28 kg/m^2^ are at increased risk of inadequate treatment, independent of the statin dose.^23^ Also, in a large-scale meta-analysis with 265 766 patients reported by Khan et al, individuals with lower BMI ( < 25 kg/m^2^) showed the greatest risk reduction in myocardial infarction, major adverse cardiovascular events, and cardiovascular mortality following LDL-lowering therapies compared to groups with greater BMI.^9^ In the current guidelines, there is no recommendation on dose adjustment based on patients’ weight or BMI. However, in a cross-sectional study on 52 916 patients from 30 countries, Ferrières et alshowed a positive correlation between BMI and prescribed daily statin intensity even after adjustment for presence of DM, cerebrovascular, ischemic heart, and peripheral artery diseases.^31^ This finding is consistent with our results, which demonstrates the better efficacy of statins in patients with lower BMI.

 In contrast, there are other investigations claiming that there is no significant association between BMI and LDL goal achievement. In a cross-sectional study on 5718 patients with stable symptomatic ASCVD treated with statins for secondary prevention, Tsai et al found no meaningful relation between the patients’ baseline BMI level and LDL goal achievements. However, patients with higher BMI were more likely not to meet the TG and HDL therapeutic goals.^32^ In another study, Bhan et al found that obese patients (BMI > 30 kg/m^2^) were more likely not to reach the therapeutic target for the cholesterol/HDL ratio. However, LDL target ( < 96.7 mg/dL) achievement was not affected by BMI.^8^ The two above-mentioned studies have some limitations and differences which may explain their inconsistency with our results. Those were cross-sectional and different groups of statins were prescribed by various doctors based on physician’s judgment and there could be considerable inter-physician variations. Also, as guidelines are mostly focused on decreasing the LDL and obesity as well-known risk factors for cardiovascular problems, obese patients tend to be prescribed higher doses of lipid-lowering agents^8^ which can bias the outcome. Furthermore, the therapeutic target was defined as LDL-C < 100 mg/dL or LDL < 96.7 mg/dL, which is directly affected by the starting value and is different from the goal we set.

 As the former treatment goals were set on LDL < 100 mg/dL and LDL < 70 mg/dL for patients at high risk and very high risk of cardiovascular disease,^33^ the effect of baseline LDL on reaching the targets was biased, because, individuals with a lower starting LDL, even after a small decrease, were considered as patients meeting the goal. However, in high baseline LDL groups, even after greater decreases, they may have not fulfilled the goal. In contrast to the prior research,^6,23^ we found a significant negative association between baseline LDL and target achievement. To the best of our knowledge, this is the first study evaluating the effect of baseline LDL on current therapeutic goal achievement success; although there are studies on small dense LDL (sd-LDL), which is the most atherogenic subclass of LDL and is believed to have an important role in the development of ASCVD.^34^ In a meta-analysis reported by Takagi et al, it was noticed that individuals with greater baseline LDL treated with rosuvastatin were more likely to show a significant reduction in sd-LDL.^35^

 In our study both moderate- and high-intensity statin therapies could significantly reduce the hs-CRP and IL-6 levels in patients with T2DM and mild hyperlipidemia. Systemic inflammation is an important precursor for atherosclerosis.^36^ CRP is the most commonly studied inflammatory marker associated with cardiovascular disease although the causality is still not proven.^37^ IL-6 is a pro-inflammatory cytokine associated with cardiovascular mortality which has major impact on acute-phase response by inducing CRP synthesis in the liver.^38,39^ The effect of statins in decreasing CRP and thus reducing cardiovascular problems is well-known^40^ but the effect is not identical for different statins and in different patients.^41,42^ In an investigation performed on patients with combined hyperlipidemia (LDL > 130 mg/dL and TG of 200 to 600 mg/dL) by Jialal et al after a 6-week statin administration, the hs-CRP level was significantly decreased in all three groups of 10 mg/d atorvastatin, 20 mg/d simvastatin, and 40 mg/d pravastatin.^43^ Soran et al reported great variability in hs-CRP response to atorvastatin and although it was reduced following daily 80 mg atorvastatin, the reduction was not statistically significant.^44^

 The mechanism of statins for decreasing CRP is controversial.^45^ In an *in vitro* study, Arnaud et al^46^ showed that statins reduce the effect of IL-6 on hepatocytes for CRP production.^46^ However, in an in*-vivo* study performed by Thongtang et al^47^, there was no significant decrease in CRP production after an 8-week daily consumption of 80 mg atorvastatin and the reduced serum CRP level resulted from increased CRP catabolism. In a systematic review and meta-analysis reported by Tabrizi et al, statins reduced the CRP and IL-6 levels significantly^48^ and decreased IL-6 could reduce the CRP production. The results of our study mostly concurred with decreasing CRP production by using statins. The subgroup with a significant decrease in hs-CRP in our study also had a reduced level of IL-6. We also believe that the reduction of IL-6 has a temporal priority to hs-CRP decrease. Rosuvastatin subgroups that had a significant decline in IL-6 levels may need a longer period of time to show hs-CRP changes. Further investigations are needed to better understand the drug mechanism.

 There are limitations applicable to this investigation. The first limitation is the small number of participants in each group which reduces the power of studying the confounders. Secondly, as all the patients had T2DM, the generalizability of the finding is limited to this specific population. Also, the drug administration period was shorter than a year which is less than the time needed to see the optimal effect of the statins.

 In conclusion, despite the general conception, moderate-intensity statins are not adequate for the majority of patients with T2DM and mild hyperlipidemia and greater numbers of patients could reach the LDL cholesterol target with high-intensity statin therapy.
